# Impact of Weight Loss on the Total Antioxidant/Oxidant Potential in Patients with Morbid Obesity—A Longitudinal Study

**DOI:** 10.3390/antiox9050376

**Published:** 2020-05-01

**Authors:** Barbara Choromańska, Piotr Myśliwiec, Magdalena Łuba, Piotr Wojskowicz, Hanna Myśliwiec, Katarzyna Choromańska, Małgorzata Żendzian-Piotrowska, Jacek Dadan, Anna Zalewska, Mateusz Maciejczyk

**Affiliations:** 1 First Department of General and Endocrine Surgery, Medical University of Bialystok, 24a M. Sklodowskiej-Curie Street, 15-276 Bialystok, Poland; piotr.mysliwiec@umb.edu.pl (P.M.); ananau@wp.pl (M.Ł.); pwojsk@wp.pl (P.W.); jacdad@poczta.onet.pl (J.D.); 2 Department of Dermatology and Venereology, Medical University of Bialystok, 14 Żurawia Street, 15-540 Bialystok, Poland; hanna.mysliwiec@umb.edu.pl; 3 Department of Oral Surgery, Medical University of Gdansk, 7 Dębinki Street, 80-211 Gdansk, Poland; kasia24_89@o2.pl; 4 Department of Hygiene, Epidemiology and Ergonomics, Medical University of Bialystok, 2c Mickiewicza Street, 15-233 Bialystok, Poland; malgorzata.zendzian-piotrowska@umb.edu.pl; 5 Experimental Dentistry Laboratory, Medical University of Bialystok, 24a M. Sklodowskiej-Curie Street, 15-274 Bialystok, Poland; anna.zalewska1@umb.edu.pl

**Keywords:** obesity, bariatric surgery, laparoscopic sleeve gastrectomy, antioxidants, total antioxidant activity

## Abstract

The assessment of total antioxidant activity seems to have a higher diagnostic value than the evaluation of individual antioxidants separately. Therefore, this is the first study to assess the total antioxidant/oxidant status in morbidly obese patients undergoing bariatric surgery. The study involved 60 patients with Class 3 obesity (BMI > 40 kg/m^2^) divided into two equal subgroups: morbidly obese patients without and with metabolic syndrome. The analyses were performed in plasma samples collected before surgery as well as 1, 3, 6, and 12 months after a laparoscopic sleeve gastrectomy. Total antioxidant capacity (TAC), ferric-reducing antioxidant power (FRAP), DPPH (2,2′-diphenyl-1-picrylhydrazyl) radical assay, and total oxidant status (TOS) were significantly higher before surgery (as compared to the healthy controls, *n* = 60) and generally decreased after bariatric treatment. Interestingly, all assessed biomarkers correlated positively with uric acid content. However, the total antioxidant/oxidant potential did not differ between obese patients without metabolic syndrome and those with both obesity and metabolic syndrome. Only DPPH differentiated the two subgroups (*p* < 0.0001; AUC 0.8) with 73% sensitivity and 77% specificity. Plasma TAC correlated positively with body mass index, waist–hip ratio, serum insulin, and uric acid. Therefore, TAC seems to be the best biomarker to assess the antioxidant status of obese patients.

## 1. Introduction

Obesity has become a worldwide health problem [1]. Numerous epidemiological studies have shown that with increasing BMI (body mass index), the risk of death goes up [1,2]. Obesity-related mortality is mainly due to a much higher incidence of cancer as well as cardiovascular and metabolic diseases [2]. Recent studies indicate that disturbances in pro-oxidant/antioxidant balance play a critical role in the pathogenesis of obesity and its related complications. Overproduction of reactive oxygen (ROS) and nitrogen (RNS) species alters cellular metabolism and signaling pathways (e.g., NF-κB, NJK, p21RAS), causing oxidative damage to lipids, proteins, and nucleic acids [3,4]. In obesity, ROS also trigger the AMP-activated protein kinase (AMPK), disrupting proliferation and apoptosis as well as insulin signaling in the target tissues [5]. Moreover, oxidative stress leads to the development of chronic inflammation in the adipose tissue [6]. 

The mainstay of obesity treatment is to increase physical activity and change dietary behaviors. Nevertheless, weight loss is difficult to maintain over a long time [7,8]. It is also insufficient in patients with morbid obesity (body mass index (BMI) > 40 kg/m^2^). Thus, the most effective method of treatment for morbid obesity is bariatric surgery [9]. Of the many surgical techniques, the most popular bariatric procedures are sleeve gastrectomy and Roux-en-Y gastric bypass [10]. Bariatric surgery has been shown not only to improve physical function and sustain long-term weight loss, but also to reduce the risk of obesity-associated diseases such as type 2 diabetes, hypertension, or cardiovascular disease [11,12,13]. However, the exact mechanism by which these changes occur is not yet known. It is therefore interesting whether the reduction of oxidative stress levels can play a role in weight loss after bariatric treatment?

The use of redox biomarkers in the diagnosis of and elucidation of the mechanisms underlying systemic diseases has recently begun to gain increasing attention. Indeed, the clinical usefulness of antioxidants has been demonstrated in the diagnosis of neurodegenerative diseases, cancers, and metabolic diseases such as insulin resistance, type 2 diabetes, non-alcoholic steatosis, hypertension, and chronic heart failure [14,15,16,17,18]. However, many compounds with antioxidant properties may have additive or synergistic effects, hence the interest in the concept of assessing the resultant antioxidant capacity of the biological system. A number of parameters are used for this purpose, including total antioxidant capacity (TAC), ferric-reducing antioxidant power (FRAP), and DPPH (2,2′-diphenyl-1-picrylhydrazyl) radical-scavenging assay. 

In our previous study, we demonstrated abnormalities of the enzymatic and non-enzymatic antioxidant barrier, which partially persist after bariatric surgery [19]. Although some of the redox parameters were normalized after the procedure and some were not, the total antioxidant/oxidant potential change in patients with morbid obesity is still unknown. Similarly, it has not been studied whether the effectiveness of the antioxidant barrier differs in obese patients without metabolic syndrome from those with both obesity and metabolic syndrome. Assuming that total antioxidant potential includes the interaction of all antioxidants (endogenous or exogenous) [20], the aim of our study was to evaluate the impact of bariatric surgery on plasma TAC, FRAP, and DPPH in morbidly obese patients before and 1, 3, 6, and 12 months after bariatric treatment. We also evaluated the total oxidant status (TOS) and calculated the oxidative stress index (OSI) to assess the redox status of obese patients.

## 2. Materials and Methods

The study involved 60 patients with Class 3 obesity (BMI > 40 kg/m^2^; women aged 28 to 56) who were treated with laparoscopic sleeve gastrectomy. The patients were classified into two subgroups depending on the diagnosis of metabolic syndrome (MS) according to the International Diabetes Federation guidelines [21]. The study subgroups consisted of morbidly obese patients without metabolic syndrome (OB; *n* = 30) and morbidly obese patients with metabolic syndrome (OB+MS; *n* = 30). The blood samples were collected before bariatric surgery (OB 0; OB+MS 0). Additionally, blood samples were taken during control visits 1 month (OB 1; OB+MS 1), 3 months (OB 3; OB+MS 3), 6 months (OB 6; OB+MS 6), and 12 months (OB 12; OB+MS 12) after bariatric treatment. In the OB+MS subgroup, thirteen patients had type 2 diabetes mellitus (T2DM) and twenty-six patients had hypertension. Morbidly obese patients had a mean weight loss of 6 ± 0.7 kg over a time interval of 10 to 30 days prior to bariatric surgery. This was associated with a low-calorie diet, which was part of the preparation for the laparoscopic sleeve gastrectomy. During this time, patients consumed a low-fat, normal-protein, and 100 g/day carbohydrate diet with a caloric content of 800–1000 kcal/day. White sugar, sweets, sweet fruits, and products containing saturated fatty acids, as well as alcohol, were eliminated from the diet. Obese patients ate five meals (three main meals and two snacks) at intervals of three hours and drank 2 L of fluids (including 1 L of water). After bariatric surgery, patients ate five balanced meals at fixed times of the day. In the first months after the surgery, the caloric content of the diet was 700–900 kcal/day. In the long term, the diet was adjusted to the patient’s age, height, and weight and was 1000–1200 kcal. The diet was enriched with calcium, vitamin B12, folic acid, and iron supplementation. 

Patients were treated at the First Department of General and Endocrine Surgery at the University Hospital in Bialystok, Poland. 

The lean group included 60 lean healthy women (BMI < 25 kg/m^2^) visiting the Specialist Dental Clinic at the Medical University of Bialystok, Poland. Only subjects within reference ranges of blood count and biochemical blood tests (K^+^, Na^+^, creatinine, INR, ALT, AST) were qualified for inclusion in the control group. The control group was adjusted according to the age and sex of patients from the study group. All the subjects were fed a balanced diet (2000 kcal; 55% carbohydrates, 30% fat, and 15% protein).

The lean and obese patients were qualified for the study based on a negative medical history with regard to metabolic diseases such as type 1 diabetes, osteoporosis, mucopolysaccharidosis, and gout; infectious diseases (HIV/AIDS, hepatitis A, B, or C), autoimmune diseases (ulcerative colitis, Hashimoto’s or Crohn’s disease), and cardiovascular (with the exception of arterial hypertension), digestive, respiratory, and genitourinary system diseases. In the three month period preceding the study, patients and healthy controls had not taken any vitamins, antioxidant supplements, nonsteroidal anti-inflammatory drugs, glucocorticosteroids, or antibiotics. The participants did not chronically drink alcohol or smoke. Patients with acute inflammatory diseases and history of malignancy were also excluded from the study.

The study was conducted in accordance with the Helsinki Declaration and Good Clinical Practice, as well as was approved by the Ethics Committee of the Medical University of Bialystok, Poland (number of permission: R-I-002/69/2012 and R-I-002/187/2017). Informed consent was given by all participants of the study.

## 3. Blood Collection

Blood samples were collected from obese and lean patients in overnight fasting state into EDTA tubes (S-Monovette SARSTEDT). In the 24 h before blood sampling, patients did not carry out intensive physical activity. All samples were centrifuged at 4000 rpm at in 4 °C for 10 min. The samples were stored at −80 °C until final analysis.

## 4. Laboratory Measurements

The laboratory parameters such as blood counts and biochemical tests were quantified using an Abbott analyzer (Abbott Diagnostics, Wiesbaden, Germany). Homeostatic model assessment index (HOMA-IR) was calculated according to Matthews et al. [22].

## 5. Redox Assays

For the redox assays, all reagents were obtained from Sigma-Aldrich (Nümbrecht, Germany/Saint Louis, MO, USA). The absorbance/fluorescence of samples was measured using a 96 well microplate reader (Infinite M200 PRO Multimode Tecan; Tecan Group Ltd., Männedorf, Switzerland). All determinations were performed in duplicate samples. The results were standardized to 1 mg of total protein. 

### 5.1. Total Antioxidant Capacity (TAC)

The level of plasma total antioxidant capacity (TAC) was assessed spectrophotometrically at 660 nm using ABTS (2,2-azinobis-3-ethylbenzothiazoline-6-sulfonic acid) radical cation and Trolox (6-hydroxy-2,5,7,8-tetramethylchroman-2-carboxylic acid) as a standard [23]. 

### 5.2. Total Oxidant Status (TOS)

The level of plasma total oxidant status (TOS) was assessed bichromatically (560/800 nm) based on the oxidation of Fe^2+^ to Fe^3+^ in the presence of the oxidants contained in the sample [24]. 

### 5.3. Oxidative Stress Index (OSI)

Oxidative stress index (OSI) was calculated as TOS to TAC ratio: OSI = TOS/TAC [24,25].

### 5.4. Radical-Scavenging Activity Assay (DPPH)

The antioxidant potential of plasma was also measured using DPPH (1,1-diphenyl-2- picrylhydrazyl) radical [26]. This compound becomes discolored in the presence of antioxidants, which was the basis for spectrophotometric measurement at 515 nm. Trolox was used as a standard. 

### 5.5. Ferric-Reducing Antioxidant Power (FRAP)

The level of ferric-reducing antioxidant power (FRAP) was determined colorimetrically based on the reduction of Fe^2+^ to Fe^3+^, resulting in the formation of a colorful ferrous tripyridyltriazine (Fe^3+^-TPTZ) complex [27]. The reaction occurs in an acidic environment. Absorbance was measured at 592 nm.

## 6. Statistical Analysis

GraphPad Prism 8.3.0 for MacOS (GraphPad Software, Inc. La Jolla, USA) was used for statistical analysis. The normality of the distribution was assessed using the Shapiro-Wilk test, while the homogeneity of the variance was checked with Levene’s test. For comparison of the quantitative variables, Kruskal-Wallis ANOVA test and Dunn’s test were used. The statistical significance level was set at *p* < 0.05. Multiplicity-adjusted *p*-values were also calculated. 

The relationship between the assessed parameters was evaluated using the Spearman rank correlation coefficient. In order to determine the diagnostic utility of plasma redox biomarkers, a receiver operating characteristic (ROC) curves were drawn, and the area under the curve (AUC) was calculated. 

The number of subjects in the groups was determined based on our previous experiment, assuming that the power of the test = 0.9.

## 7. Results

### 7.1. Clinical and Laboratory Parameters

Before and after bariatric surgery, BMI and WHR (waist–hip ratio) were significantly greater in both morbidly obese groups than the lean controls. Glucose, insulin, HOMA-IR, and total cholesterol as well as CRP and white blood count were higher, whereas HDL levels were diminished in both studied obese groups compared to the controls. Additionally, the BMI and WHR decreased after bariatric surgery in both morbidly obese groups, as assessed 6 and 12 months after bariatric surgery (Table 1).

### 7.2. Total Antioxidant Capacity (TAC)

**Figure 1:**
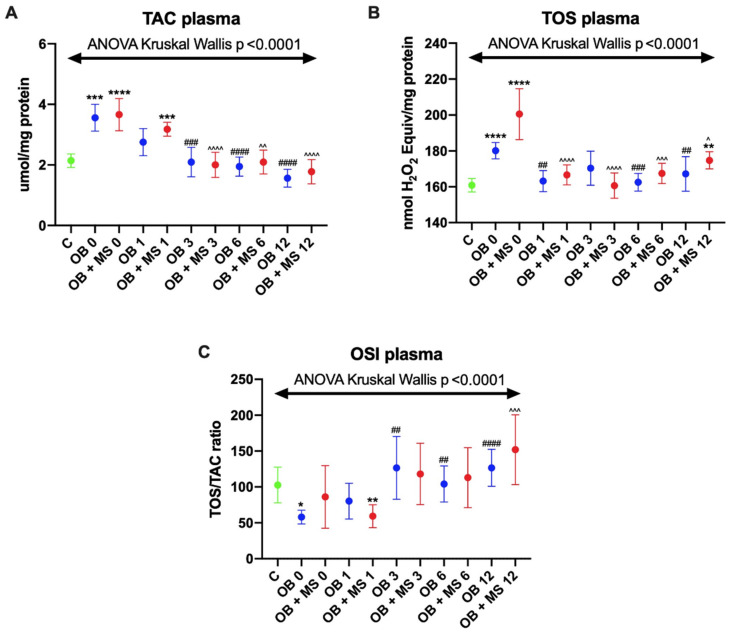
Plasma TAC (**A**), TOS (**B**), and OSI (**C**) values of the control (C), morbid obesity without metabolic syndrome (OB), and morbid obesity with metabolic syndrome (OB+MS) groups. Results are shown as median with 95% Cl. * *p* < 0.05, ** *p* < 0.01, *** *p* < 0.001, **** *p* < 0.0001 indicate significant differences from the control; ## *p* < 0.01, ### *p* < 0.001, #### *p* < 0.0001 indicate significant differences from the OB 0 patients; ^ *p* < 0.05, ^^ *p* < 0.01, ^^^ *p* < 0.001, ^^^^ *p* < 0.0001 indicate significant differences from the OB+MS 0. TAC: total antioxidant capacity; TOS: total oxidant status; OSI: oxidative status index; OB 0 and OB+MS 0: before bariatric surgery; and OB 1 and OB+MS 1: 1 month, OB 3 and OB+MS 3: 3 months, OB 6 and OB+MS 6: 6 months, and OB 12 and OB+MS 12: 12 months after laparoscopic sleeve gastrectomy.

We noticed significantly higher plasma TAC in both obese groups: OB 0 (+62%, *p* = 0.0001) and OB+MS 0 (+68%, *p* < 0.0001) in comparison with the controls. Moreover, in morbidly obese patients with metabolic syndrome TAC was still significantly elevated 1 month after the bariatric surgery (OB 1: +52%, *p* = 0.0007). Further on, we observed that TAC values in both obese groups gradually decreased after obesity surgery: OB 3 (−42%, *p* = 0.0007), OB 6 (−50%, *p* < 0.0001), OB 12 (−59%, *p* < 0.0001), OB+MS 3 (−47%, *p* < 0.0001), OB+MS 6 (−46%, *p* < 0.0019), and OB+MS 12 (−52%, *p* < 0.0001), as compared to OB 0 and OB+MS 0 patients (Figure 1A).

### 7.3. Total Oxidant Status (TOS)

Plasma TOS of OB 0 (+12%, *p* < 0.0001), OB 12 (+10%, *p* < 0.0001), and OB+MS 0 (+19%, *p* < 0.0001) patients had significantly greater values compared to the lean subjects. Furthermore, after bariatric treatment, plasma TOS significantly diminished at OB 1 (−9%, *p* = 0.0038), OB 6 (−10%, *p* = 0.0007), and OB 12 (−9%, *p* = 0.0087) compared with OB 0 as well as OB+MS 1 (−14%, *p* < 0.0001), OB+MS 3 (−15%, *p* < 0.0001), OB+MS 6 (−12%, *p* = 0.0001), and OB+MS 12 (−10%, *p* = 0.0406) compared with OB+MS 0 (Figure 1B).

### 7.4. Oxidative Stress Index (OSI)

We observed decreased OSI in plasma at OB 0 (−33%, *p* = 0.0168) and OB+MS 1 (−32%, *p* = 0.003) compared to the controls. Interestingly, after bariatric surgery, OSI markedly increased in both morbidly obese groups: OB 3 (+66%, *p* = 0.097), OB 6 (+88%, *p* = 0.0022), OB 12 (+129%, *p* < 0.0001), and OB+MS 12 (+84%, *p* = 0.003) as compared to OB 0 and OB+MS 0, respectively (Figure 1C).

### 7.5. 2,2′-Diphenyl-1-Picrylhydrazyl Radical (DPPH) Assay

**Figure 2:**
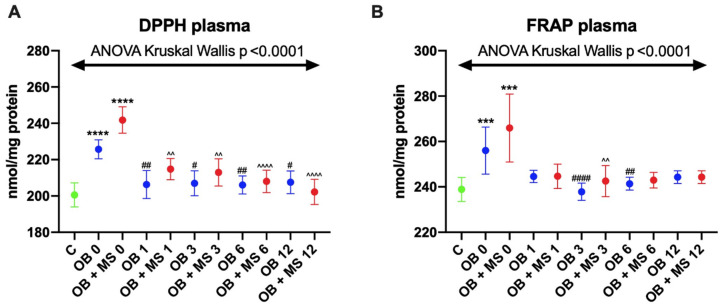
Plasma DPPH (**A**) and FRAP (**B**) of the control (C), morbid obesity without metabolic syndrome (OB), and morbid obesity with metabolic syndrome (OB+MS) groups. Results are shown as median with 95% Cl. *** *p* < 0.001, **** *p* < 0.0001 indicate significant differences from the control; # *p* < 0.05, ## *p* < 0.01, #### *p* < 0.0001 indicate significant differences from OB 0 patients; ^^ *p* < 0.01, ^^^^ *p* < 0.0001 indicate significant differences from the OB+MS 0 patients. DPPH: 2,2′-diphenyl-1-picrylhydrazyl radical; FRAP: ferric-reducing antioxidant power; OB 0 and OB+MS 0, before bariatric surgery; and OB 1 and OB+MS 1: 1 month, OB 3 and OB+MS 3: 3 months, OB 6 and OB+MS 6: 6 months, and OB 12 and OB+MS 12: 12 months after laparoscopic sleeve gastrectomy.

We found markedly higher plasma DPPH values in both obese groups: OB 0 (+12%, *p* < 0.0001) and OB+MS 0 (+22%, *p* < 0.0001) in comparison with the lean controls. Moreover, plasma DPPH decreased after bariatric treatment in OB 1 (−8%, *p* = 0.0062), OB 3 (−8%, *p* = 0.0158), OB 6 (−10%, *p* = 0.0014), OB 12 (−8%, *p* = 0.0235), OB+MS 1 (−13%, *p* = 0.0024), OB+MS 3 (−14%, *p* = 0.0011), OB+MS 6 (−15%, *p* < 0.0001), and OB+MS 12 (−18%, *p* < 0.0001) subgroups as respectively compared to OB 0 and OB+MS 0 patients (Figure 2A).

### 7.6. Ferric-Reducing Antioxidant Power (FRAP)

Plasma FRAP of OB 0 (+6%, *p* = 0.0002) and OB+MS 0 (+6%, *p* = 0.0003) subgroups was significantly increased as compared to the lean controls. However, after bariatric treatment, we noticed that plasma FRAP significantly decreased at OB 3 (−6%, *p* < 0.0001) and OB 6 (−5%, *p* = 0.0086) compared with OB 0, as well as at OB+MS 3 (−6%, *p* = 0.0012) compared with OB+MS 0 patients (Figure 2B).

### 7.7. Correlations

**Figure 3:**
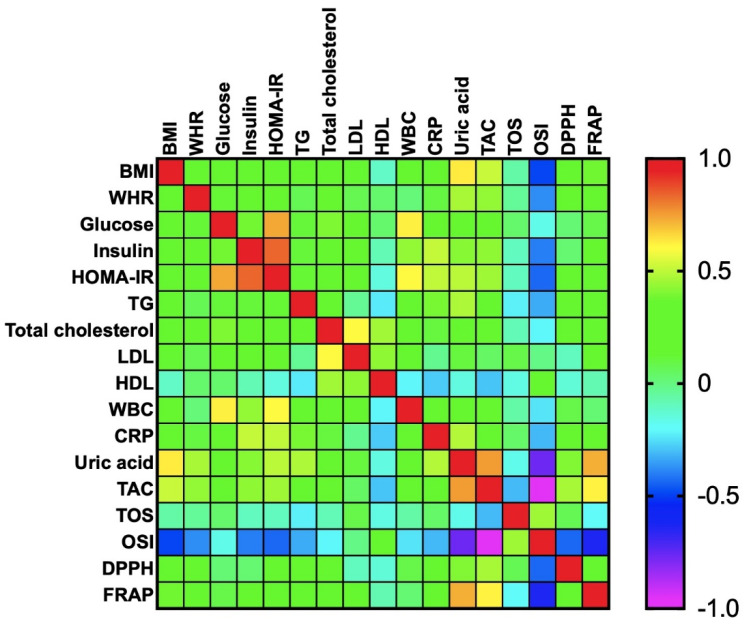
Correlations between the analyzed redox biomarkers and clinical parameters in morbidly obese patients. BMI: body mass index; WHR: waist-hip ratio; HOMA-IR: homeostatic model assessment of insulin resistance; TG: triacylglycerol; LDL: low-density lipoprotein; HDL: high-density lipoprotein; WBC: white blood cell count; CRP: C-reactive protein; TAC: total antioxidant capacity; TOS: total oxidant status; OSI: oxidative status index; DPPH: 2,2′-diphenyl-1-picrylhydrazyl radical; FRAP: ferric reducing antioxidant power.

**Figure 4:**
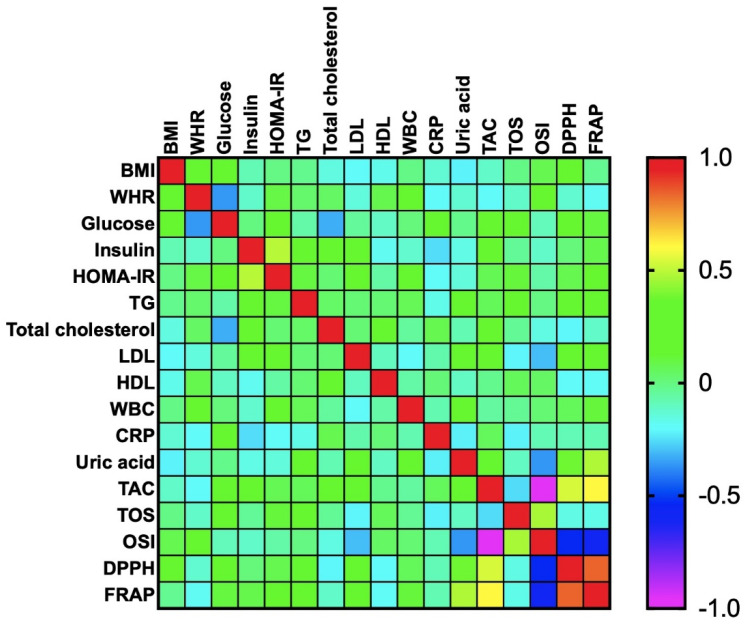
Correlations between the analyzed redox biomarkers and clinical parameters in lean patients. BMI: body mass index; WHR: waist-hip ratio; HOMA-IR: homeostatic model assessment of insulin resistance; TG: triacylglycerol; LDL: low-density lipoprotein; HDL: high-density lipoprotein; WBC: white blood cell count; CRP: C-reactive protein; TAC: total antioxidant capacity; TOS: total oxidant status; OSI: oxidative status index; DPPH: 2,2′-diphenyl-1-picrylhydrazyl radical; FRAP: ferric-reducing antioxidant power.

Correlations between the analyzed redox biomarkers and clinical parameters are presented in heat maps (Figure 3 and Figure 4). The exact values of correlation coefficient and significance level are given in the Supplementary Material (Tables S1–S4).

In morbidly obese patients, serum UA was positively correlated with BMI (R = 0.628; *p* < 0.0001), WHR (R = 0.467; *p* < 0.0001), HOMA-IR (R = 0.492; *p* < 0.0001), triglycerides (R = 0.473; *p* < 0.0001), and CRP (R = 0.485; *p* < 0.0001).

Plasma TAC was positively associated with BMI (R = 0.515; *p* < 0.0001), WHR (R = 0.435; *p* = 0.001), HOMA-IR (R = 0.453; *p* < 0.0001), serum insulin (R = 0.427; *p* = 0.001), and serum UA (R = 0.752; *p* < 0.0001). A positive correlation was also observed between plasma DPPH and serum UA (R = 0.407; *p* = 0.001), as well as plasma FRAP and serum UA (R = 0.724; *p* < 0.0001). A negative correlation was demonstrated between plasma OSI and serum UA (R = −0.768; *p* < 0.0001), OSI, and BMI (R = −0.492; *p* < 0.0001), as well as OSI and HOMA-IR (R = −0.436; *p* = 0.001).

Additionally, we found a positive correlation between plasma TAC and FRAP (R = 0.62; *p* < 0.0001) as well as TAC and DPPH (R = 0.464; *p* < 0.0001). However, OSI was negatively correlated with plasma TAC (R = −0.975; *p* < 0.0001), DPPH (R = −0.436; *p* = 0.001), and FRAP (R = −0.635; *p* < 0.0001). On the other hand, OSI was positively associated with plasma TOS (R = 0.451; *p* < 0.0001) (Figure 3).

In healthy controls, we found a positive correlation between plasma FRAP and serum UA (R = 0.476; *p* < 0.0001). 

Plasma TAC was associated with plasma DPPH (R = 0.537; *p* < 0.0001) and FRAP (R = 0.611; *p* < 0.0001). However, OSI was negatively correlated with plasma TAC (R = - 0.965; *p* < 0.0001), DPPH (R = −0.536; *p* < 0.0001), and FRAP (R = −0.593; *p* < 0.0001). A positive correlation was also shown between OSI and TOS (R = 0.467; *p* < 0.0001). We also noticed a positive correlation between plasma DPPH and FRAP (R = 0.841; *p* < 0.0001) (Figure 4).

### 7.8. ROC Analysis

**Figure 5:**
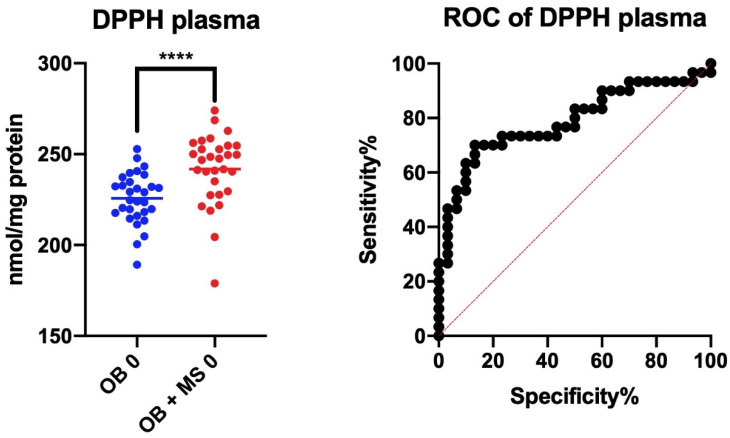
Area under the curve (AUC) of DPPH (2,2′-diphenyl-1-picrylhydrazyl) radical assay between the obese patients without metabolic syndrome (OB) and obese patients with metabolic syndrome before bariatric surgery (OB+MS). **** *p* < 0.0001 indicates significant difference from OB 0.

We checked whether the assessed redox biomarkers differentiated morbid obesity patients from those with both morbid obesity and metabolic syndrome. The results of the ROC analysis are presented in Table 2. Interestingly, we showed a very high diagnostic value for the evaluation of plasma DPPH. This parameter differentiated patients with morbid obesity from obese patients with MS with high sensitivity (73%) and specificity (77%) (AUC 0.79; *p* < 0.0001) (Figure 5). 

## 8. Discussion

In recent years, much attention has been paid to explaining the pathogenesis of morbid obesity. The World Health Organization (WHO) estimates that at least 3 million people worldwide die each year from obesity-related complications [2]. Numerous studies suggest that metabolic diseases accompanying obesity can be caused not only by a higher accumulation of bioactive lipids [28,29], but also by disturbances in pro-oxidant/antioxidant balance [19,30]. Indeed, both increase and decrease of the antioxidant barrier have been observed in morbidly obese patients [28,31,32]. However, it is still unclear whether bariatric surgery improves the redox homeostasis of obese patients. 

Indeed, there have been only a few studies of the impact of surgical treatment on pro-oxidant/antioxidant homeostasis. In our previous research, we found disturbances in enzymatic and non-enzymatic antioxidant status, which partially normalized after bariatric surgery [19]. Serum superoxide dismutase (SOD) and plasma-reduced glutathione (GSH) were significantly diminished in both obese groups before bariatric surgery, while uric acid (UA) and glutathione disulfide (GSSG) were statistically higher. Nevertheless, after bariatric surgery, the activity of SOD increased and concentration of UA decreased only in obese patients without metabolic syndrome. Given the interactions between antioxidants and their different contributions to the pathology of obesity, the present study is the first to assess the impact of bariatric surgery on the total antioxidant/oxidant potential in obese individuals. We also compared the antioxidant barrier between patients with morbid obesity to those with both obesity and metabolic syndrome.

Since antioxidants can have an additive or synergistic effect on each other, the assessment of the total antioxidant activity seems to have a higher diagnostic value than the evaluation of individual antioxidants separately [33]. The total antioxidant potential, often referred as “total antiradical activity”, represents the overall ability of the biological system to scavenge oxygen free radicals. There are several methods for measuring total antioxidant activity. In this study, we used the most common: TAC, FRAP, and DPPH. They are based on the same principle; oxidants initiate a reaction that can be observed colorimetrically. The presence of antioxidants in the sample delays the substrate’s oxidation, which is proportional to the antioxidant content in the bioliquid. Nevertheless, the contribution of individual antioxidants to the total antioxidant potential is different [34,35]. For example, the share of GSH in total antioxidant potential will be much lower in the FRAP assay than in the other methods (TAC or DPPH) [26]. 

We showed that the total antioxidant (↑TAC, ↑FRAP, ↑DPPH) and oxidant (↑TOS) potential were significantly higher before surgery and generally decreased after bariatric treatment. Using the ROC analysis, we showed that plasma DPPH and TOS differentiate patients with obesity from subjects with both obesity and metabolic syndrome.

Total antioxidant potential and oxidative stress are closely interlinked. Indeed, decreased efficiency of antioxidant systems predisposes to oxidation of proteins, lipids, and DNA, which is a direct cause of oxidative stress. The change in the concentration of plasma antioxidants also allows the redox imbalance of many systemic diseases to be assessed. It may also be a the therapeutic strategies when the oxidative–reduction balance is shifted to the oxidation side [36].

Consequently, enhanced TAC, FRAP, and DPPH levels may suggest an increased ability to remove free radicals, and thus effective protection against oxidative stress in obese patients. This is not surprising, as antioxidants are the main defensive system against ROS/RNS overproduction. However, higher oxidative damage to proteins, lipids, and DNA was observed in patients with morbid obesity. Increased levels of oxidative modification products were found not only in plasma but also in skeletal muscles, adipose tissue, liver, and salivary glands [19,37,38,39]. How then to explain the increase in total antioxidant potential in obese patients? Up to 70–80% of the total antioxidant potential represents UA, which in physiological concentrations is the most important plasma antioxidant. However, this compound may also generate free radicals (inter alia in reaction with peroxynitrite). This happens with very high concentrations of UA [40]. Indeed, under these conditions, UA has strong pro-oxidant and pro-inflammatory properties. It has been shown that UA stimulates the production of ROS and the synthesis of cytokines such as interleukin 1β and -6 (IL-1β, IL-6), MCP-1 (monocyte chemotactic protein 1), and TNF-α [40,41,42]. In our study, this was confirmed by a positive relationship between the serum UA and CRP level. In addition, the serum UA concentration correlated positively with plasma TAC (R = 0.752; *p* < 0.0001), FRAP (R = 0.724; *p* < 0.0001), and DPPH (R = 0.407; *p* < 0.0001). Therefore, an increase in the total antioxidant potential may result from enhanced plasma UA content. Interestingly, hyperuricemia seems to be not only a predictive factor, but also an independent risk factor for hypertension, metabolic syndrome, and cardiovascular disease [41,42]. 

What about other antioxidants affecting the total antioxidant potential? It can be assumed that antioxidant systems are activated in the early stages of the disease. With the long-term development of obesity, the source of antioxidants gradually depletes, leading to a reduced activity of the antioxidant barrier [19,43]. However, it can be assumed that hyperuricemia is mainly responsible for disturbances in plasma redox status of obese patients. Therefore, in order to improve redox homeostasis of obese patients, the UA concentration should be normalized.

Although we did not directly evaluate the rate of ROS production, increased TOS level (before surgery) may indicate an intensification of pro-oxidative processes in obese patients. Indeed, TOS describes the total amount of oxidants in the biological system and is one of the indicators of oxidative stress [24]. These observations were also confirmed by a decrease in the oxidative stress index (compared before and 1 month after the surgery). It is well known that an important source of ROS in obesity is the adipose tissue. Indeed, adipokines secreted by the adipose tissue activate the transcription factor NF-κB, which impairs the bioavailability of NO and increases the production of free radicals [44,45]. Moreover, higher levels of fatty acids and glucose lead to endothelial cell damage and activation of the pro-inflammatory cascade. This also increases ROS formation, similarly to enhanced NADPH oxidase (NOX) activity [44,45]. 

Strengthening of the antioxidant barrier in obese patients results undoubtedly from the improvement of metabolic status after bariatric surgery (↓BMI, ↓WHR, ↓UA, ↓LDL, ↓TG, ↓glucose, ↓insulin, and ↓HOMA-IR). An increased supply of food rich in antioxidants can also lead to weight loss and improved redox homeostasis [46,47]. Therefore, it is not surprising that better effects of bariatric surgery were achieved in obese patients without metabolic syndrome. In this group, abnormalities of pro-oxidant/antioxidant systems began to normalize in the first months after the bariatric treatment. Furthermore, OSI increased significantly only from 6 (OB group) and 12 months (OB+MS group) after bariatric surgery (compared to pre-operative values). Enhanced intensity of oxidative processes in patients with metabolic syndrome was also indicated by the increase in TOS at the end of the experiment (compared to healthy controls). Indeed, despite a huge weight loss 1 year after bariatric surgery, the OB patients were overweight, while the OB+MS patients were Class 1 obese. It is therefore likely that weight loss and improvement of the antioxidant barrier are still not enough to balance the oxidation processes. 

The total antioxidant/oxidant potential is increasingly used in the diagnosis of various systemic diseases. Indeed, the diagnostic usefulness of salivary/plasma/urine TAC and TOS has been demonstrated in patients with dementia, Alzheimer’s disease, chronic kidney disease, and psoriasis [48,49,50,51,52]. Nevertheless, in our study, the total antioxidant/oxidant potential did not differ in patients with morbid obesity compared to those with both obesity and metabolic syndrome. Only plasma DPPH differentiated the two groups, with 73% sensitivity and 77% specificity (AUC 0.8; *p* < 0.0001). Significant differences were also noted for plasma TOS (AUC 0.7; *p* = 0.002). However, the ideal biomarker should also correlate with the degree of disease. Therefore, the best parameter to assess the antioxidant status of obese patients could be the total antioxidant capacity (TAC). Indeed, the plasma TAC correlated positively with BMI, WHR, HOMA-IR, serum insulin, and uric acid. However, such relationships were not observed in the control group. To assess the diagnostic utility of plasma TAC, further research is needed on a larger population of obese patients.

Finally, it is also worth noting the limitations of our research. Despite a carefully selected study group and controls (in terms of accompanying diseases and age), we are not able to eliminate the impact of hypotensive/antidiabetic drugs on the assessed redox biomarkers. Although patients received detailed dietary recommendations, we did not conduct a dietary interview and cannot assess the impact of nutrition on the total antioxidant potential. Another limitation of the study is that the evaluation of redox status was conducted in women only. However, as we showed previously, plasma and erythrocyte antioxidant activity are not sex-specific [53]. It is also necessary to confirm the diagnostic usefulness of the analyzed redox biomarkers on a larger group of subjects.

## 9. Conclusions

In summary, the total antioxidant/oxidant potential of obese patients was significantly higher before bariatric surgery. Generally, it did not differ between obese patients without metabolic syndrome and those with metabolic syndrome, with the exception of DDPH. It normalized after bariatric treatment and depended mainly on the uric acid content. Plasma TAC seems to be the best biomarker to assess the antioxidant status of obese patients.

## Figures and Tables

**Table 1:** Clinical and laboratory parameters of the control group (C), patients with morbid obesity without metabolic syndrome (OB), and patients with both morbid obesity and metabolic syndrome (OB+MS). Results are shown as median (minimum and maximum); * *p* < 0.05 indicates significant differences from the control; # *p* < 0.05 indicates significant differences from the OB 0 patients; ^ *p* < 0.05 indicates significant differences from the OB+MS 0 patients. BMI: body mass index; WHR: waist–hip ratio; HOMA-IR: homeostatic model assessment of insulin resistance; LDL: low-density lipoprotein; TG: triacylglycerol; HDL: high-density lipoprotein; ALT: alanine transaminase; AST: aspartate transaminase; CRP: C-reactive protein; WBC: white blood cell count; RBC: red blood cell count; HGB: hemoglobin; PLT: platelet count; OB 0 and OB+MS 0: before bariatric surgery; OB 1 and OB+MS 1: 1 month, OB 3 and OB+MS 3: 3 months; OB 6 and OB+MS 6: 6 months, and OB 12 and OB+MS 12: 12 months after laparoscopic sleeve gastrectomy.

Characteristics	C	OB 0	OB+MS 0	OB 1	OB+MS 1	OB 3	OB+MS 3	OB 6	OB+MS 6	OB 12	OB+MS 12
**Age**	45 (28–56)	39 (31–52)	48 (28–56)								
**BMI (kg/m**^**2**^)	23 (17–25)	45 * (40–55)	47 * (41–61)	41 * (30–49)	42 * (33–57)	37 * # (28–47)	39 * (29–50)	33 * # (25–42)	34 * ^ (26–48)	29 # (23–38)	31 * ^ (23–40)
**WHR**	0.71 (0.65–0.75)	0.96 * (0.81–1)	0.98 * (0.84–1.2)	0.96 * (0.74–1)	0.99 * (0.87–1.2)	0.94 * (0.8–1)	0.98 * (0.83–1.1)	0.93 * (0.81–0.98)	0.96 * (0.87–1.1)	0.91 * (0.8–0.98)	0.93 * ^ (0.85–0.99)
**Glucose (mg/dL)**	78 (57–98)	98 * (76–122)	106 * (89–264)	93 * (69–115)	99 * (75–147)	88 (60–142)	98 * (79–116)	85 (68–109)	96 * (81–157)	84 # (73–111)	90 * ^ (77–99)
**Insulin (μIU/mL)**	7.6 (6.4–9.7)	18 * (12–39)	22 * (9.3–41)	11 * # (4–22)	15 * (5.8–35)	7.8 # (2.7–16)	11 * ^ (3.6–22)	8.7 # (4.1–12)	8.8 ^ (4.5–17)	7.8 # (3–15)	8.5 ^ (4.5–9.9)
**HOMA–IR**	1.4 (1.2–2.1)	4.2 * (2.6–9.3)	5.8 * (3.1–15)	2.7 * # (0.73–5.6)	3.8 * (1.3–8.6)	1.9 # (0.46–3.1)	2.5 * ^ (0.95–4.4)	1.8 # (0.76–2.9)	2 * ^ (1.1–3.8)	1.7 # (0.59–3.5)	1.9 ^ (1.1–2.2)
**Cholesterol (mg/dL)**	175 (158–194)	195 * (147–231)	211 * (167–268)	176 (124–210)	192 * (126–235)	176 (120–217)	181 ^ (120–237)	178 (114–245)	188 * (148–264)	176 (114–231)	174 ^ (138–295)
**LDL (mg/dL)**	118 (111–123)	125 (102–159)	144 * (122–181)	109 (83–153)	122 ^ (66–183)	109 (69–159)	116 ^ (50–189)	109 (61–166)	110 ^ (83–157)	111 # (59–134)	102 * ^ (64–164)
**TG (mg/dL)**	135 (119–150)	127 (62–197)	150 (104–289)	108 * (66–209)	136 (75–367)	102 * (46–182)	137 (78–240)	92 * (48–182)	130 (89–214)	90 * # (36–182)	102 * ^ (59–146)
**HDL (mg/dL)**	60 (45–70)	48 * (33–62)	46 * (31–72)	44 * (30–106)	45 * (31–68)	47 * (27–141)	49 * (35–69)	50 * (35–72)	53 (35–86)	53 * (38–81)	56 ^ (43–70)
**ALT (IU/L)**	24 (8–35)	25 (6–93)	27 (12–54)	25 (6–52)	28 (14–56)	20 (6–43)	24 (13–59)	17 (6–51)	21 (12–31)	18 (8–37)	19 (8–43)
**AST (IU/L)**	24 (16–36)	19 (13–44)	21 (14–50)	22 (14–98)	27 (14–85)	19 (12–36)	21 (13–43)	17 (9–48)	20 (12–43)	18 (12–52)	19 (12–37)
**CRP (mg/L)**	5.6 (4.7–6.5)	9.1 * (1.5–28)	12 * (5.3–18)	5.6 (0.5–26)	8.4 * (1.5–14)	6.1 (0.3–17)	7.1 (0.6–17)	5.1 (0.3–20)	6.1 ^ (0.5–11)	4.9 # (0.2–7.8)	5.5 ^ (1.2–16)
**Uric acid (mg/dL)**	4 (2.3–6.4)	6 * (4.3–13)	7.1 * (4.6–13)	5.2 * (3.7–11)	6.1 * (3.8–9.5)	4.9 # (3–8.5)	5.4 * (3.4–8.2)	4.5 # (2.4–6.4)	5 * ^ (3.9–7.1)	4.2 # (2.3–5.7)	4.5 ^ (3.3–6.3)
**Urea (mg/dL)**	24 (17–41)	28 (18–38)	29 * (18–60)	24 # (7–34)	27 (20–47)	22 # (7–45)	26 (17–41)	22 (9–43)	26 (19–51)	22 (11–47)	25 (18–55)
**WBC (10** ^**3**^ **/μL)**	7.4 (4.4–9.7)	8.3 * (6.1–12)	9.5 * (5.9–13)	6.6 (4.6–9.6)	7.2 (5–13)	5.8 (4.6–11)	6.9 (3.9–12)	6 (4.8–9.3)	7.2 (4.4–11)	5.9 (4.1–8.5)	7 (4.2–10)
**RBC (10** ^**6**^ **/μL)**	4.7 (3.9–5.3)	4.6 (3.5–5.8)	4.6 (4–5.5)	4.8 (4.1–6)	4.8 (4.3–6)	4.6 (4.1–5.1)	4.8 (4.1–5.5)	4.6 (4–5.7)	4.8 (4–5.7)	4.6 (4.2–5.4)	4.5 (3.3–5.7)
**HGB (g/dL)**	14 (12–16)	13 (11–15)	13 (12–16)	13 (10–16)	14 (11–17)	13 (12–16)	13 (11–16)	13 (10–15)	13 (11–16)	14 (9–16)	14 (9–16)
**PLT (10** ^**3**^ **/μL)**	289 (259–315)	262 (141–417)	258 (183–418)	261 (121–412)	229 * (128–405)	266 (130–312)	247 * (131–345)	292 (188–425)	252 (167–375)	201 * (130–345)	224 * (164–378)

**Table 2:** Area under the curve (AUC) of total antioxidant potential between the obese patients without metabolic syndrome (OB) and obese patients with metabolic syndrome before bariatric surgery (OB+MS). TAC: total antioxidant capacity; TOS: total oxidant status; OSI: oxidative status index; DPPH: 2,2′-diphenyl-1-picrylhydrazyl radical; FRAP: ferric-reducing antioxidant power.

Parameter	Area	95% Confidence Interval	*p*-Value	Cut Off	Sensitivity (%)	95% CI	Specificity (%)	95% CI
TAC	0.53	0.3851 to 0.6816	0.657	>3.529	56.67	39.20% to 72.62%	53.33	36.14% to 69.77%
TOS	0.74	0.6158 to 0.8647	0.002	>184.8	66.67	48.78% to 80.77%	62.07	44.00% to 77.31%
OSI	0.54	0.3871 to 0.6865	0.628	>52.96	60	42.32% to 75.41%	55.17	37.55% to 71.59%
DPPH	0.79	0.6669 to 0.9064	<0.0001	>234.9	73.33	55.55% to 85.82%	76.67	59.07% to 88.21%
FRAP	0.53	0.3855 to 0.6834	0.647	>251.6	56.67	39.20% to 72.62%	53.33	36.14% to 69.77%

## Supplementary Materials

The following are available online at https://www.mdpi.com/2076-3921/9/5/376/s1, Table S1: *r* value of correlation between the analyzed redox biomarkers and clinical parameters in morbidly obese patients. Table S2: *p*-values of correlation between the analyzed redox biomarkers and clinical parameters in morbidly obese patients. Table S3: *r*-values of correlation between the analyzed redox biomarkers and clinical parameters in the control group. Table S4: *p*-values of correlation between the analyzed redox biomarkers and clinical parameters in the control group.
